# Thermos-responsive hydrogel system encapsulated engineered exosomes attenuate inflammation and oxidative damage in acute spinal cord injury

**DOI:** 10.3389/fbioe.2023.1216878

**Published:** 2023-08-08

**Authors:** Yu Xiao, Xinmei Hu, Peng Jiang, Zhongquan Qi

**Affiliations:** Medical College of Guangxi University, Nanning, Guangxi, China

**Keywords:** exosomes, PLGA-PEG-PLGA, miR-138-5p, NLRP3, Nrf2, SCI

## Abstract

**Introduction:** Spinal cord injury (SCI) is a serious and disabling condition, and the effectiveness of conventional treatment is limited, such as supportive treatment and emergency surgery. Exosomes derived from umbilical cord mesenchymal stem cells (UCMSC-Exos) have potential therapeutic effects on SCI but are limited by delivery efficiency. Our study aimed to further investigate the therapeutic effects of miR-138-modified UCMSC-exosomes (Exos-138) following SCI.

**Methods:** We developed an injectable triblock polymer of polyglycolic acid copolymer and polyethylene glycol (PLGA-PEG-PLGA)-loaded temperature-sensitive hydrogel of miR-138-modified stem cell exosomes and characterised its biocompatibility *in vitro*. In Sprague-Dawley rats with SCI, the hydrogel was injected into the injury site, behavioural scores were measured, and pathological analysis was conducted postoperatively to assess neurological recovery.

**Results:** *In vitro*, our data demonstrated that miR-138-5p-modified UCMSC-Exos can reduce inflammation levels in BV-2 cells through the NLRP3-caspase1 signalling pathway and reduce neuronal apoptosis by downregulating intracellular reactive oxygen species levels through the Nrf2-keap1 signalling cascade. The results of *in vivo* experiments showed that the P-Exos-138 hydrogel promoted neurological recovery in rats with SCI.

**Discussion:** Our study explored a novel exosome delivery system that can be a potential therapeutic strategy for SCI. Our study, currently, has theoretical value; however, it can serve as a basis for further investigations on the treatment approaches at various stages of SCI development in inflammation-dependent injury of the central nervous system.

## Introduction

Spinal cord injury (SCI) is serious and devastating, and is often caused by falls from height or traffic accidents, resulting in reduced quality of life and increased social burden (Barnabé-Heider and Frisén, 2008). After the initial mechanical injury, SCIs can develop secondary pathologies such as neuroinflammation and oxidative damage (Sykova et al., 2021). In the late stages of injury, the inhibitory microenvironment and scar formation at the injury site interfere with axonal regeneration, rendering drug therapy minimally effective (Kwiecien et al., 2020; Qian et al., 2022).

In recent years, stem cell transplantation technology has received considerable attention from researchers as a promising strategy for the treatment of SCI, including bone marrow mesenchymal stem cells (MSCs), embryonic stem cells, and umbilical cord MSCs (UCMSCs) (Gao et al., 2020). In contrast to the results of preclinical studies, stem cell transplantation technology has many obstacles in clinical application, such as its possible tumorigenicity and its inability to survive for a long time in an inhibitory microenvironment (Liu et al., 2021). With the development of regenerative medicine, scientists have gradually recognised that exosomes derived from stem cells play a major role in regenerative programmes after tissue damage (Bobis-Wozowicz et al., 2015). Exosomes are extracellular vesicles with a diameter ranging from 30 to 150 nm, rich in various miRNAs, RNAs, proteins, and other biological cargo (Elsherbini and Bieberich, 2018). Benefiting from the penetration ability of exosomes, it can be applied in the treatment of central nervous system diseases (Zheng et al., 2020). Exosomes can transfer components that, when bound to target cells, mediate intercellular communication by delivering their internal nucleic acids and proteins (Krämer-Albers and Hill, 2016). These molecules can modulate relevant signalling pathways by binding to intracellular targets, ultimately affecting the physiological processes of the target cells (Gu et al., 2020). In central nervous system (CNS) diseases, exosomes can improve CNS pathologies by reducing neuroinflammation, promoting the regenerative capacity of neurons, and modulating local immune responses (Hartmann et al., 2017; Li et al., 2019).

miRNAs in exosomes are important components of functional substances and are thought to play an important role in reducing exosome-induced neuronal apoptosis. For example, the injection of miR-133-modified exosomes into SCI rats can protect damaged neurons by activating the STAT3, ERK1/2, and CREB signalling pathways and improve the recovery of motor function in the hind limbs of SCI rats (Li et al., 2018). In addition, exosomes from miR-126-modified MSCs can reduce neuronal apoptosis and promote functional regeneration after SCI (Huang J. et al., 2020).

miR-138-5p, a member of the miRNA family, is closely associated with several human diseases, especially inflammatory diseases (including many types of cancers), in which miR-138-5p is downregulated in hypoxia/reoxygenation-induced acute myocardial infarction models (Wang et al., 2021). In contrast, miR-138-5p can regulate the inflammatory response in early acute lung injury by inhibiting NLRP3 activation (Luo et al., 2021). Neuroinflammation is mainly mediated by microglia and phagocytes (de Araújo Boleti et al., 2019). A previous study showed that lncRNA H19 promotes the inflammatory response induced by cerebral ischaemia-reperfusion injury by regulating the miR-138-5p/p65 axis (Li et al., 2020). Meanwhile, Xiaojin Feng et al. provided evidence for the anti-neuroinflammatory effects of miR-138-5p; they found that the activation of the NLRP3/caspase-1 cascade was likely associated with the downregulation of miR-138-5p expression levels in a rat model of LPS-induced cognitive impairment, ultimately causing hippocampal neuroinflammation in rats (Feng et al., 2021a).

Furthermore, new biomaterials such as hydrogels or collagenous bio-scaffolds have been used for SCI repair (Xu et al., 2022; Zhou et al., 2022). Hydrogel scaffolds can be used as support materials for transplanted cells to fill the SCI site, whereas drug-loaded hydrogels can release drugs uniformly in the affected area to reduce the local inflammatory response so that axons can regenerate in an orderly manner (Gao et al., 2022). Unlike natural biological scaffolds, the triblock polymer of the polyglycolic acid copolymer and polyethylene glycol (PLGA-PEG-PLGA) is a synthetic temperature-sensitive hydrogel. It is commonly used in the fields of anti-tumour and anti-arthritis owing to its excellent biocompatibility and *in situ* slow-release effects (Chen M. et al., 2021; Yan et al., 2021). Several recent studies have shown that stem cell-derived exosomes can bind to hydrogel bio-scaffolds to promote axonal regeneration and modulate angiogenesis in SCI (Fan et al., 2022; Mu et al., 2022).

In our study, we used an injectable temperature-sensitive hydrogel to deliver UCMSC-derived exosomes (Exos) and Exos-138. Further, we used H_2_O_2_-stimulated BV-2 microglia to mimic the cell injury scenario *in vitro* and aimed to explore the anti-inflammatory and antioxidant roles of Exos-138 by modulating the NLRP3-caspase1 pathway and Nrf2-keap1 signalling axis. *In vivo*, we explored the effects of PLGA-PEG-PLGA-loaded Exos-138 on functional recovery in a rat SCI model.

## Materials and methods

### Reagents and chemicals

The red fluorescent cell linker kit (PKH26) and cell counting kit-8 (CCK-8) were purchased from Sigma–Aldrich (St. Louis, MO, United States). Beyotime Biotechnology (Jiangsu, China) provided the reactive oxygen species (ROS) assay kit and RIPA lysis buffer. Human umbilical cord mesenchymal stem cells (hUCMSCs) and BV2 microglial cell lines were obtained from Procell Co. Ltd. (Wuhan, China). Dulbecco’s Modified Eagle Medium (DMEM) and foetal bovine serum (FBS) were obtained from Gibco (Scoresby, VIC, Australia). TSG101 and CD63 antibodies were purchased from Abcam (Cambridge, United Kingdom), whereas Nrf2, Keap1, HO-1, NLRP3, and Caspase-1 antibodies were purchased from Cell Signalling Technology (Danvers, MA, United States). The Prime Script RT Reagent Kit and SYBR Green PCR master mix were obtained from Takara Bio Inc. (Takara, Japan), while the Exo-Fect transfection kit was purchased from System Biosciences (Palo Alto, United States). MiR-138-5p mimics were obtained from Anornor Biotechnology (Guangzhou, China).

### Cell culture

For hUCMSCs, the cells were proliferated in human stem cell serum-free medium supplemented with penicillin and streptomycin solutions. After the fifth passaging generation of hUCMSCs reached 80% confluence, cells were cultured for 48 h using exosome-free FBS and the medium was collected. For BV2 microglia, cells were cultured in DMEM supplemented with 10% FBS and maintained at 37°C and 5% CO_2_.

### Preparation and identification of exosomes derived from hUCMSCs

hUCMSC-exosomes were collected from the medium by differential centrifugation. Briefly, the completed medium was centrifuged at 800 *g* for 10 min and 3,000 ×*g* for 30 min at 4°C. After further centrifugation at 10,000 ×*g* for 1 h at 4°C, the supernatant was filtered using a 0.22 μm filter (Millipore, United States) to remove cells and debris. The supernatant was ultracentrifuged twice at 120,000 ×*g* for 2 h. The hUCMSC-exosome pellets were resuspended in 200 μL phosphate-buffered saline (PBS) for subsequent cell and animal experiments. Next, to identify exosomes, the formation of Exos was verified using transmission electron microscopy (TEM, HT-7700, Japan) and nanoparticle tracking analysis (NTA; ZetaView, Malvern, United Kingdom). Specific exosome surface markers (CD63 and TSG101) were identified using Western blotting.

### Loading of miR-138-5p into exosomes

Exo-Fect ™ was used to load miR-138-5p according to the manufacturer’s instructions. First, miR-138-5p mimic loading was carried out by incubating Exos (1×10^9^ particles) at 37°C for 10 min with Exo-Fect (10 μL, to a final volume of 150 μL). Then, all samples were mixed with Exo-Quick reagent in a 1:5 (v/v) ratio on ice for 30 min and finally, centrifuged at 13,000 ×*g* for 5 min at 4°C. The supernatant and pellet were separated and Exos-138 was collected and resuspended in PBS for cell culture. The FAM-miR-138-5p-modified exosomes (FAM-Exos-138) were co-cultured with 1×10^6^/well BV2 cells in 6-well plates for 12 h. The fluorescence of BV2 cells was observed using a Cytation 5 Cell Imaging Multimode Reader (BioTek LionheartLX) and total cellular RNA was extracted for qPCR experiments.

### Exos-138 uptake assay

Briefly, a PKH26 dye working solution at a concentration of 4 μM was prepared by diluting 1 μL of dye storage solution with 250 μL of diluent C reagent (UR52303, Umibio, China). Two hundred and 50 μL of PKH26 dye working solution was added to 250 μL of Exos-138 solution, mixed thoroughly, and incubated at 37°C for 5 min. The reaction between Exos-138 and the PKH26 dye working solution was terminated by adding 500 μL of 1% bovine serum albumin (BSA) for 2 min. The volume of the mixture was then increased to 25 mL, and the mixture was centrifuged at 120,000 ×*g* for 120 min. The supernatant was discarded, and the residue (PKH26-Exos-138) was suspended in PBS. PKH26-Exos-138 cells were co-cultured with 1×10^6^/well BV2 cells in 6-well plates for 12 h. The fluorescence of the BV2 cells was observed using a Cytation 5 Cell Imaging Multimode Reader (BioTek LionheartLX).

### Preparation and characterisation of P-Exos-138

A commercially available injectable hydrogel was used in this study (Xi’an Qiyue Biotechnology, Xian, China). PLGA-PEG-PLGA was dissolved at a 1:4 (w/w) ratio in PBS containing Exos or Exos-138, respectively. PLGA-PEG-PLGA (20 wt%) was dissolved in water at room temperature (25°C), the morphology of P-Exos-138 was verified using transmission electron microscopy, and the concentration of released Exos-138 was evaluated using the bicinchoninic acid reagent test kit. The sol-gel transition temperature of the P-Exos-138 copolymer was determined by an inverted rotation test (Huang C. et al., 2020).

Cytotoxicity assays were performed using the CCK-8 assay. The BV2 cells were seeded overnight in 96-well plates to ensure adherence. Different complexes (P-Gel, P-Exos, or P-Exos-138) were added to transwell inserts and co-cultured with the cells for 48 h. Subsequently, CCK-8 reagent was added to each well, and the absorbance at 450 nm was recorded using a microplate reader (Multiscan Spectrum; Thermo Labsystem, Chantilly, VA).

### CCK-8 assay

BV2 cells were cultured at a density of 6×10^3^ cells/well in the plates overnight. The following day, the cells were treated with P-Exos-138 for 48 h or with different concentrations of H_2_O_2_ (100 nM, 200 nM, or 300 nM) for 6 h. The CCK-8 kit was used, and the cells were incubated at 37°C for 2 h before the absorbance at 450 nm was measured using the microplate reader.

### Intracellular ROS detection

Briefly, BV2 cells were cultured with complete medium overnight for adherence and were stimulated with serum-free medium for 1 h, pre-treated with Exos or Exos-138 (10^8^ particles/mL) for 12 h, and then stimulated with 200 μM H_2_O_2_ for 6 h in 12 mm glass cell culture chambers (ThermoFisher, Scoresby, VIC, Australia). Then, DCFH-DA was used to stain the cells for 30 min under the conditions of 37°C and 5% CO_2_. Intracellular ROS production was determined using a ROS detection kit. The fluorescence intensity was measured using a Cytation 5 Cell Imaging Multimode Reader (BioTek LionheartLX) and analysed using the ImageJ software.

The spinal cords of all groups were collected *in vivo* and frozen sections were prepared. A frozen section ROS detection kit (BioRab Technology, Beijing, China) was used. The fluorescence was detected at 525 nm using a Nikon Eclipse Ti-SR microscope (Nikon, Japan).

### Dual-luciferase reporter gene assay

We used the Dual-Luciferase Reporter Assay Kit (Promega, Madison, WI, United States) to verify the predicted potential target gene of miR-138-5p. Briefly, 293T cells were seeded at 5×10^4^ cells/well in 24-well plates, and the wild-type (WT) or mutant (MUT) NLRP3 3ʹ-UTR luciferase reporter constructs were co-transfected into 293 T cells with miR-138-5p mimic or miR-138-5p mimic NC using Lipofectamine 2000 (Invitrogen, CA, United States). After 6 h of transfection, the cells were collected and examined using a dual-luciferase reporter assay system (Promega, Madison, WI, United States).

### Quantitative reverse transcriptase PCR

BV2 cells were seeded at 1×10^5^ cells/well into 6-well plates with medium. These cells were pre-treated with Exos or Exos-138 for 12 h and then stimulated with LPS (1 μg/mL) for 12 h. Total RNA was purified using TRIzol reagent (Thermo Fisher, Scoresby, VIC Australia). To determine miR-138-5p expression, the miRNA Tailing Reaction First Strand cDNA Synthesis kit was used to synthesise miRNA First Strand cDNA, following the manufacturer’s protocols. For detection of other mRNAs such as IL-1Β, IL-18, and TNF-α, RevertAid First Strand cDNA Synthesis Kit (ThermoFisher, Scoresby, VIC, Australia) was utilised to synthesise single-stranded cDNA, which was then used for qPCR, and the reaction was performed using SYBR Green PCR MasterMix (ThermoFisher, Scoresby, VIC, Australia). β-actin and U6 were used as internal controls for mRNA and miR-138-5p, respectively. The expression levels were calculated using the 2^−ΔΔCT^ method. Primers used are listed in Table 1.

**TABLE 1:** Speciffc primers sequences of mRNAs.

Gene	Primer sequences (5ʹ-3ʹ)
*miR-138-5p*	F: AGC​TGG​TGT​TGT​GAA​TCA​GG
*IL-1β*	F: TGA​AAA​CAC​AGA​AGT​AAC​GTC​CG
	R: CCC​AGG​AGG​AAA​TTG​TAA​TGG​GA
*IL-6*	F: GGC​GGA​TCG​GAT​GTT​GTG​AT
	R: GGA​CCC​CAG​ACA​ATC​GGT​TG
*TNF-α*	F: CTG​GAT​GTC​AAT​CAA​CAA​TGG​GA
	R: ACT​AGG​GTG​TGA​GTG​TTT​TCT​GT
*iNOS*	F: ACA​TCG​ACC​CGT​CCA​CAG​TAT
	R: CAG​AGG​GGT​AGG​CTT​GTC​TC
*IL-18*	F: AGC​AGT​CCC​AAC​TAA​GCA​GTA
	R: CAG​CCA​GTA​GAG​GAT​GCT​GA
*IL-10*	F: AGG​ATG​CAC​ATC​AAA​AGG​CTT
	R: GGC​CTC​GGT​TAG​GAA​GGA​TAC
*Arg-1*	F: ACA​TTG​GCT​TGC​GAG​ACG​TA
	R: ATC​ACC​TTG​CCA​ATC​CCC​AG
*Gapdh*	F: AAT​GGA​TTT​GGA​CGC​ATT​GGT
	R: TTT​GCA​CTG​GTA​CGT​GTT​GAT

Abbreviations: F, forward; R, reverse.

### Western blot

For pathway studies, BV2 cells were cultured in 6-well plates at 1×10^5^ cells/well overnight for adherence. The cells were serum-starved for 2 h and pre-treated with Exos or Exos-138 for 12 h before stimulation with 200 μM H_2_O_2_ for 6 h. To explore the expression of NLRP3 and Caspase-1, BV2 cells were seeded at 1×10^5^ cells/well in a 6-well plate. Before stimulation with LPS for 24 h, the cells were pre-treated with Exos or Exos-138. After reaching this time point, exosomes or cell lysates were collected. Whole-cell lysates were separated by SDS-PAGE and transferred onto nitrocellulose membranes. Unspecific binding was blocked with 10% BSA for 1 h, and the membranes were incubated overnight with appropriate primary antibodies at 4°C. The membranes were then washed with tris-buffered saline (TBS)-Tween (TBST) three times before being incubated with the appropriate IRDye fluorescently labelled secondary antibodies for 1.5 h. The protein bands were visualised using a LI-COR Odyssey SA Infrared Imaging Scanner (LI-COR Biosciences), and the relative protein expression was quantified based on densitometric analysis using the associated software.

### Rat SCI model establishment and hydrogel delivery

All experimental procedures were performed in accordance with the guidelines of the Animal Studies Committee of Guangxi University. Ten-week-old female Sprague-Dawley rats purchased from Changsha Tianqin Biotechnology Co., Ltd. (Changsha, China) were randomly divided into five groups (*n* = 6), namely, the SCI, sham, P-Gel, P-Exos, and P-Exos-138 groups. Rats were anaesthetised with 10% chloral hydrate after fasting for 6 h, and T10 laminectomy was performed after dissection of the paravertebral muscles. The spinal cord was fully exposed and compressed using a vascular clamp (30 g; Oscar, Shanghai, China) for 1 min. In the sham group, laminectomy was performed without spinal cord compression injury. The hydrogel or hydrogel–exosome mixture was injected into the surface of the spinal cord. The rats were then placed under a heating lamp for 2 min to accelerate hydrogel coagulation. Subsequently, the muscle and skin were sutured, and all rats were placed in a cage where they could move freely and ingest disinfectant pellets and purified water for recovery. Postoperative treatments included injection of penicillin solution (4 × 10^6^ units per rat) and squeezing of the rat bladder to facilitate manual urination twice a day.

### Locomotion recovery assessment

Basso, Beattie, and Bresnahan (BBB) scoring and footprint analysis was used to assess motor recovery after SCI in different groups of rats. Each group of rats was assessed at 1-, 7-, 14-, 21-, and 28-day post-injury, with scores ranging from 0 (complete paralysis) to 21 (normal movement). The scoring measures were obtained by three experienced researchers who were blinded to the study groups. On day 28 post-injury, a footprint analysis was performed for each group of rats. Briefly, the forelimbs of the animals were immersed in red dye and the hindlimbs were immersed in blue dye, and the trajectories left by the rats on the paper were analysed.

### Enzyme-linked immunosorbent assay (ELISA) of spinal cord inflammatory factors

Rats were sacrificed 7 days after modelling, and the area of the spinal cord encompassing 0.5 cm from the epicentre of the injured tissue was cut in each direction. The tissues were placed in lysis buffer at 4°C to prepare 10% tissue homogenate, and the supernatant was collected after centrifugation at 1,500 ×*g* for 10 min. The levels of TNF-α, IL-1β, and IL-6 in each group’s SCI area were measured according to the instructions of the ELISA kit (Elabscience Biotechnology Co., Ltd., Wuhan, China).

### Tissue preparation, histopathological analysis, and immunofluorescence staining

Rats were anaesthetised with 10% chloral hydrate, and the area of the spinal cord encompassing 0.5 cm from the epicentre of the injured tissue was cut in each direction. For histopathological analysis, tissues were fixed overnight in 4% paraformaldehyde, embedded in paraffin, and cut into 5 μm-thick longitudinal sections. After the tissue slices were dewaxed and dehydrated with gradient alcohol, haematoxylin-eosin (HE) and tar purple were used to stain the spinal cord tissues. The areas of retained spinal cord tissue and number of motoneurons were observed under a microscope (BX53, Olympus, Japan). Spinal cords were collected, and frozen slices were prepared using a freezing microtome (Leica, Germany) for immunofluorescence analysis. Frozen slices were blocked with 10% BSA and then incubated with anti-NF200 and anti-GFAP primary antibodies overnight at 4°C, followed by incubation with a secondary antibody for 1 h at 37°C. After thorough washing, the nuclei were stained using DAPI, and a panoramic viewer (Pannoramic DESK, 3DHISTECH’s Slide Converter, Magyarorszag) was used to obtain fluorescence images.

### Immunofluorescence

BV2 cells were cultured with complete medium in 6-well plates overnight to adhere. Before stimulation with 1 μg/mL LPS for 12 h, the cells were pre-treated with Exos or Exos-138. The cells were fixed in 4% paraformaldehyde and unspecific binding was blocked with 3% BSA in PBS for 1.5 h prior. They were then incubated overnight at 4°C with primary antibodies: Iba1 (1:500, Abcam, United Kingdom), iNOS (1:500, Proteintech, China), CD206 (1:500, Proteintech, China) and after washed thoroughly, the nuclei were stained using DAPI. Finally, images were captured by a Cytation 5 Cell Imaging Multimode Reader and analyzed using ImageJ software.

### Statistical analysis

Data are presented as the mean ± standard deviation of at least three independent experiments. For the comparison between two groups, a Student’s t-test was used, and for the comparison between three or more groups, analysis of variance tests were used, complemented with Dunnett’s method for multiple comparisons. Statistical significance was set at *p* ≤ 0.05. All statistical analyses were performed using GraphPad Prism 7.0.

## Results and discussion

### Preparation and identification of Exos-138

**FIGURE 1:**
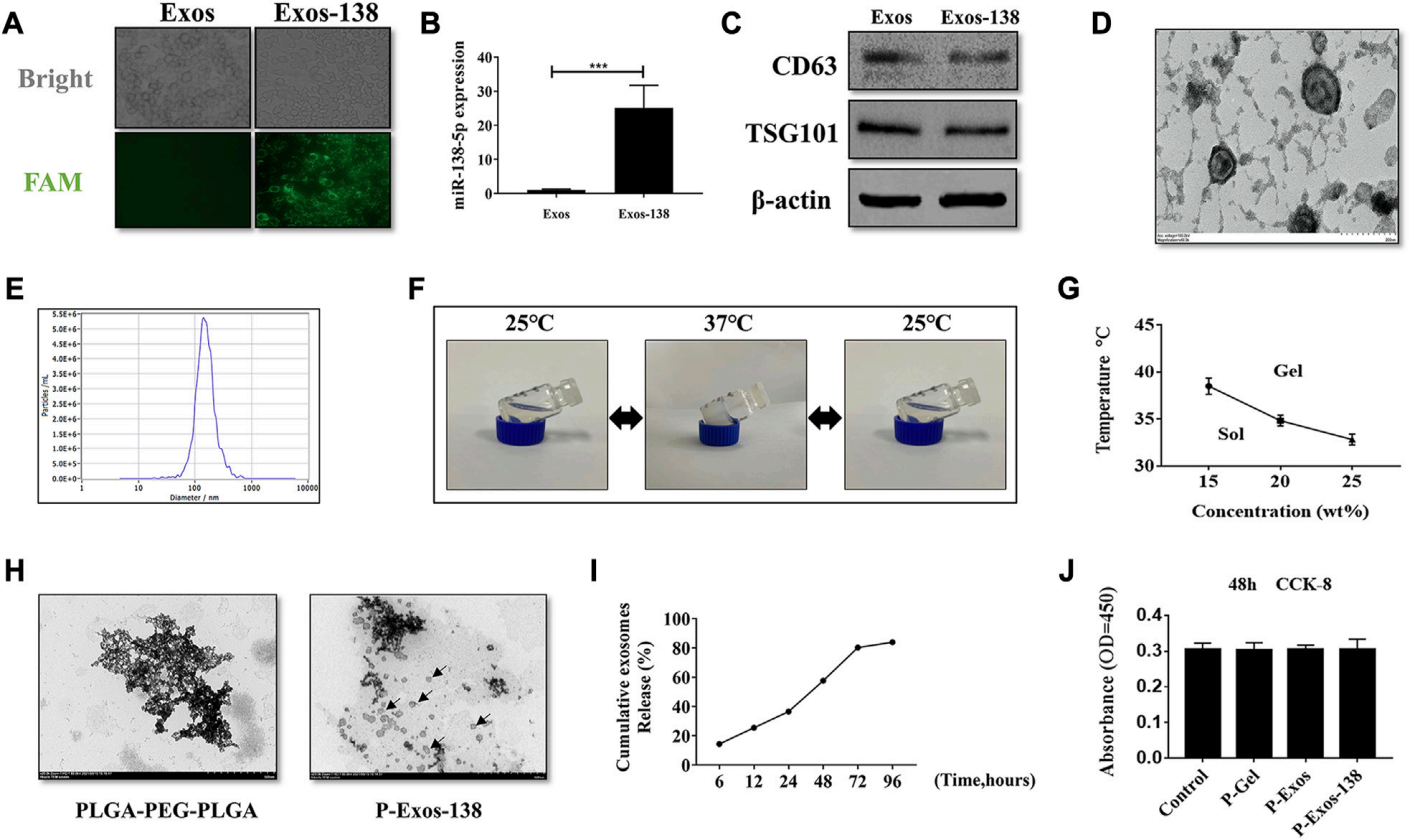
Preparation of Exos-138 and characterization of P-Exos-138 hydrogels. **(A)** Representative fluorescence images of BV-2 cells were incubated with Exos or Exos-138. **(B)** miR-138-5p expression level in BV-2 cells following co-culture with Exos or Exos-138. **(C)** Verification of expression of surface markers, including CD63 and TSG101, on Exos and Exos-138 by Western blot. **(D)** Representative transmission electron micrographs of Exos-138 showing cup-shaped morphology, scale bar = 200 nm. **(E)** Nanoparticle tracking analysis showing the size distribution of Exos-138. **(F)** Inverted tube method to measure gelation temperature. **(G)** The gelation temperature of 15 wt%, 20 wt%, and 25 wt% PLGA-PEG-PLGA hydrogels. **(H)** Representative transmission electron micrographs of PLGA-PEG-PLGA and P-Exos-138, scale bar = 500 nm. **(I)** Percentage cumulative exosome release from P-Exos-138. **(J)** CCK-8 assay to examine the cytotoxicity of PLGA-PEG-PLGA to BV-2 cells. The values presented are the means ± SD, *p ≤ 0.05, **p ≤ 0.01, ***p ≤ 0.001.

Several studies have shown that miR-138-5p becomes downregulated in the spinal cord during the days that follow the injury (Yunta et al., 2012; Maza et al., 2022). We measured the miR-138-5p expression in spinal cord tissue using RT-qPCR and confirmed the significant downregulation of miR-138-5p at 3 dpi (Supplementary Figure S1A). miR-138-5p modification was performed using exosomes derived from human UCMSCs. de Abreu et al. (2021) compared different methods for loading exogenous miRNA into exosomes, and indicates that utilizing the Exo-Fect kit to load exogenous miRNA into exosomes is the most promising method. Therefore, in our study, we prepared Exos-138 using the Exo-Fect kit. Adding FAM-miR-138-5p mimics modified Exos in BV-2 microglia, and observing the cells under fluorescence microscopy showed that miR-138-5p was successfully loaded into Exo (Figure 1A). In addition, the expression of miR-138-5p was significantly increased in cells cultured with Exos-138 (Figure 1B). Next, we identified Exos-138 using immunoblotting. Expression of the Exos-specific proteins CD63 and TSG101 was observed in Exos and Exos-138 (Figure 1C). Meanwhile, Exos-138 showed a typical cup-like structure (Figure 1D), and the NTA results showed that the particle diameter of Exos-138 was 144.2 nm (Figure 1E). The above results indicate that Exos-138 was successfully prepared and retained its exosomal characteristics.

### Characterisation of P-Exos-138 hydrogels

The phase transition temperature of polymers is important for *in vivo* applications; therefore, we tested the state of P-Exos-138 at different temperatures using the test tube inversion method. When the temperature dropped to room temperature (25°C), the hydrogel changed from gel to sol. Conversely, when the temperature rose to 37°C, the hydrogel changed from sol to gel (Figure 1F). The solidification temperature of the gel significantly decreased as the mass fraction increased (Figure 1G). Considering the internal temperature of 37°C–38°C in rats, we chose 20 wt% of the hydrogel as the exosome carrier. Transmission electron microscopy results showed the scattered presence of Exos-138 in the PLGA-PEG-PLGA particles (Figure 1H). As the hydrogel degraded, its encapsulated exosomes were released, and we further measured the release profile of exosomes, as shown in Figure 1I, where the hydrogel was able to release approximately 80% of the exosomes after 3 days. As a biomaterial, PLGA-PEG-PLGA, widely used for local chemotherapy drug delivery, and has demonstrated its non-toxic to low-toxic characteristics in various organs of experimental animals. (Ma et al., 2014; Gu et al., 2022). In several studies, PLGA-PEG-PLGA has been utilized as a drug carrier for central nervous system (CNS) diseases, also demonstrating its biocompatibility and non-toxic nature (Zheng et al., 2021; Lin et al., 2022). In this study, we evaluated the effects of P-Gel, P-Exos, and P-Exos-138 on microglial activity using the CCK8 assay, and the results showed no significant cytotoxic effects (Figure 1J). These results demonstrate that the system shows excellent cytocompatibility and potential applications *in vivo*.

### BV-2 microglia uptake of Exos-138 and inhibition of ROS accumulation *in vitro*

**FIGURE 2:**
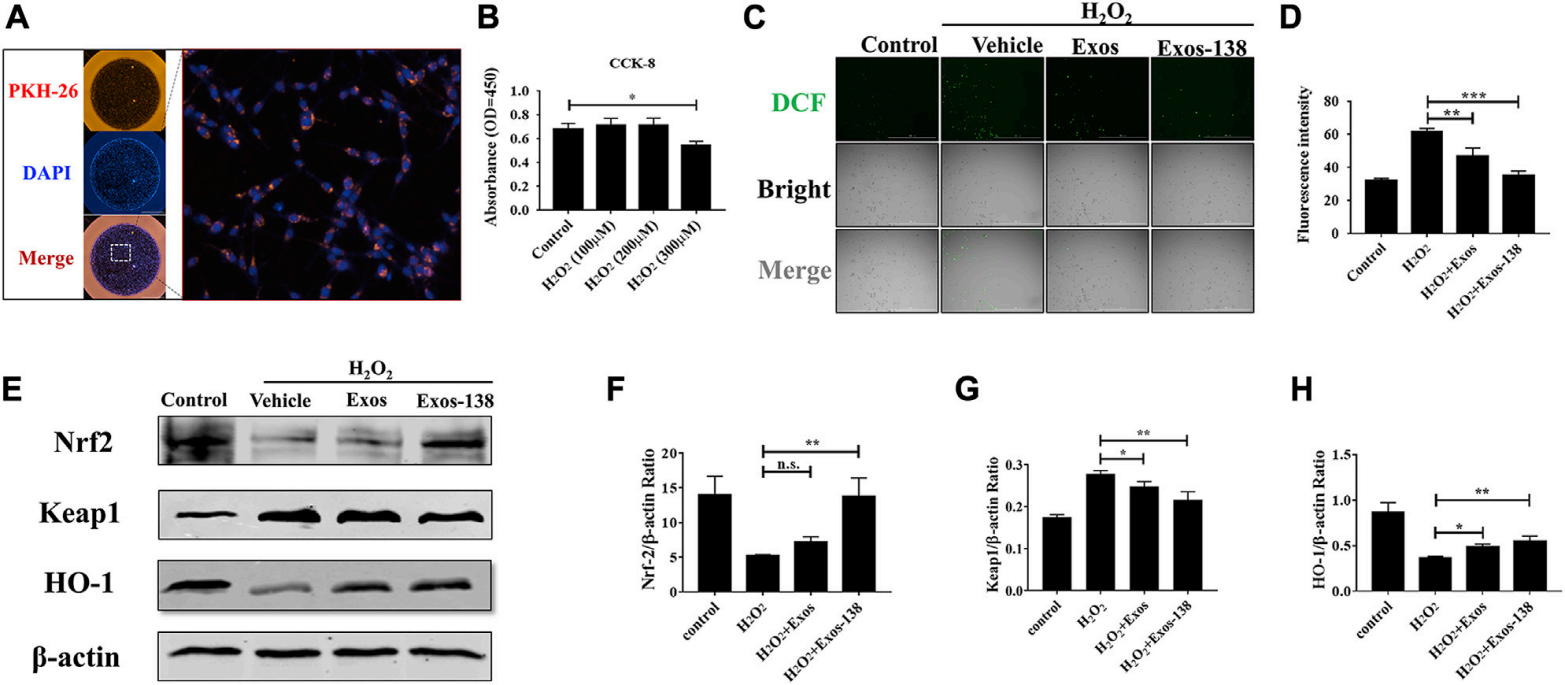
Exos-138 was internalized into BV-2 cells and inhibited Accumulation *in vitro*. **(A)** The uptake of PKH-26-labeled Exos-138 into BV-2 microglia. **(B)** CCK-8 assay to test the cell viability of BV-2 cells at various concentrations of H_2_O_2_. **(C)** Exos and Exos-138 suppressed the ROS production induced by H_2_O_2_, scale bar = 200 μm. **(D)** Quantification analysis of the average fluorescence intensity of DCFH-DA (+) cells per field of view. **(E–H)** Western blotting analysis of the protein levels of Nrf2, keap1, and HO-1 in H_2_O_2_ stimulated BV-2 cells and semi-quantification of the data. The values presented are the means ± SD, *p ≤ 0.05, **p ≤ 0.01, ***p ≤ 0.001.

To determine the effect of Exos-138 on oxidative stress in microglia, we examined the uptake of Exos-138 by microglia. As shown in Figure 2A, PKH-26-labelled Exos-138 was present in the perinuclear region of microglia after 12 h of incubation. Next, we assessed whether Exos-138 could reduce ROS levels in microglia. We exposed BV-2 microglia to a certain concentration of H_2_O_2_, and the results showed that the viability of microglia significantly decreased 6 h after 300 μM H_2_O_2_ exposure (Figure 2B); therefore, we selected 200 μM H_2_O_2_ as an inducer of oxidative stress in microglia.

The protective effect of Exos-138 against oxidative stress was detected by observing the DCFH-DA in microglia. Figures 2C, D shows that intracellular ROS levels were significantly reduced (indicating stronger antioxidant effects) in Exos-138 group than in the Exos group. Moreover, activation of the Nrf2-keap1 signalling cascade largely rescued cellular oxidative damage (Guo et al., 2010; Mao et al., 2010); therefore, we measured the effect of Exos-138 on the Nrf2-keap1 signalling cascade in the H_2_O_2_ -induced BV-2 cell injury model. We found that Exos-138 significantly increased the expression of Nrf2 protein in BV-2 cells and decreased the expression level of keap1 (Figures 2E–H). As a transcription factor, Nrf2 regulates the expression of downstream antioxidant enzymes in response to intracellular ROS accumulation, whereas Exos-138 significantly increases the expression level of HO-1, an antioxidant enzyme regulated by Nrf2. These findings collectively demonstrate that Exos-138 can be internalized by microglia to effectively reduce ROS production in BV-2 cells through the activation of the Nrf2-mediated oxidative stress pathway. The ability of Exos-138 to attenuate oxidative stress in microglia suggests its potential as a therapeutic approach for neuroinflammatory conditions associated with oxidative damage.

However, it is important to acknowledge the limitations of our study. Our study focused on a specific aspect of oxidative stress and the Nrf2-Keap1 pathway. Future investigations should explore other pathways and molecular mechanisms involved in the antioxidant effects of Exos-138 to gain a more comprehensive understanding of its therapeutic efficacy.

### Exos-138 inhibits LPS-induced M1 polarisation of microglia

**FIGURE 3:**
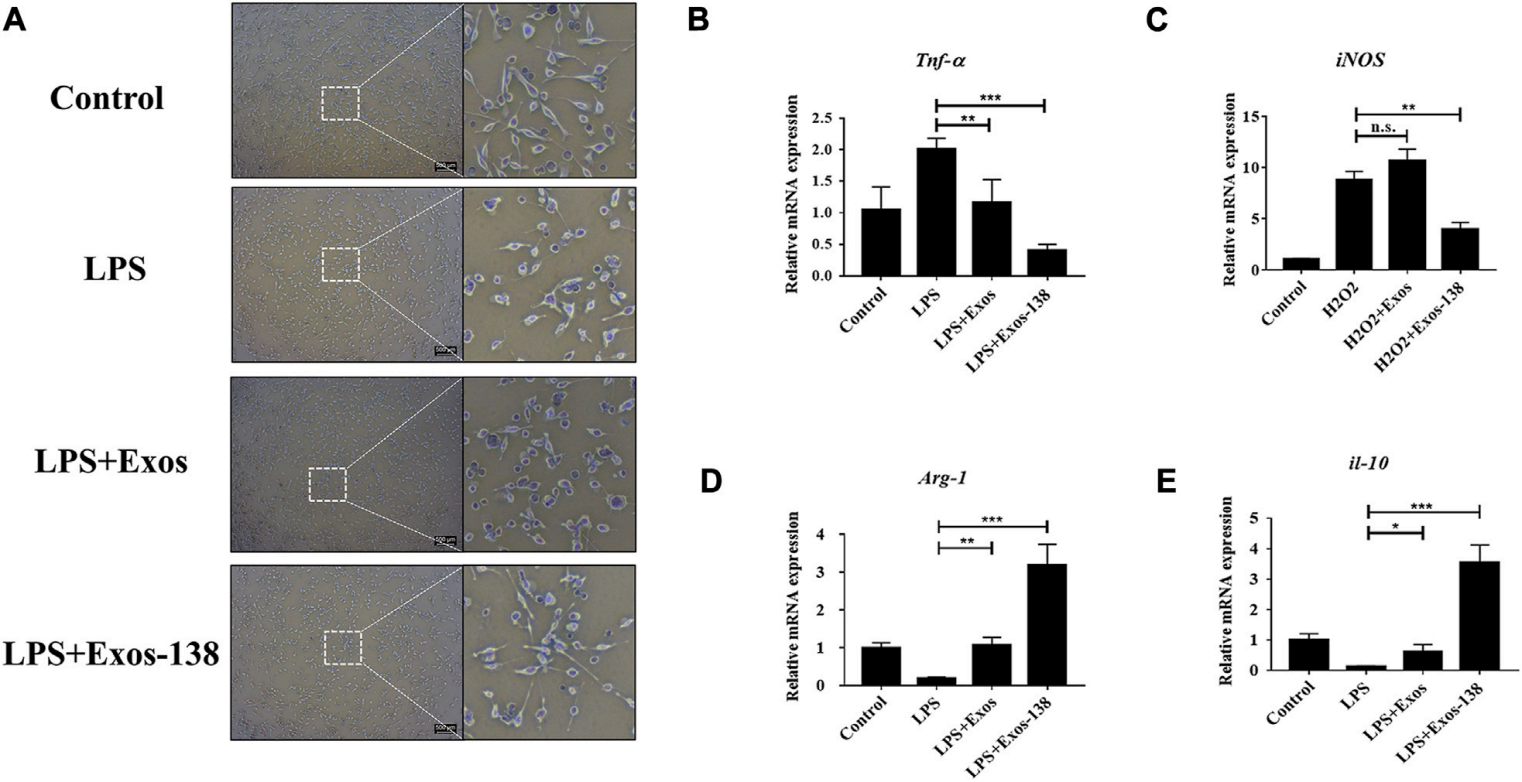
Exos-138 inhibits LPS-induced M1 polarization of microglia. **(A)** Morphological change of BV-2 microglia with LPS plus Exos or Exos-138 treatment for 24 h, scale bar = 200 μm. **(B–E)** Relative mRNA levels of pro-inflammatory factors including iNOS and Tnf-α, and anti-inflammatory factors including Arg1 and il-10. The values presented are the means ± SD, *p ≤ 0.05, **p ≤ 0.01, ***p ≤ 0.001.

Microglia respond to injury stimuli and pathogen-associated molecular patterns through various surface receptors, resulting in an overactive inflammatory state that can exacerbate the secondary inflammatory response following neurological injury (Rodríguez-Gómez et al., 2020). Activated microglia are a major source of ROS that cause further oxidative damage, such as neurodegeneration and impairment of mitochondrial function (Akbar et al., 2016). To investigate the effects of the engineered exosomes on microglial polarisation, we activated BV2 microglia using LPS to simulate the neuroinflammatory pathological state after SCI. Similar to previous studies (Zheng et al., 2021), our results showed that BV2 microglia showed amoeboid-like changes after 24 h of LPS stimulation, whereas pre-treatment with Exos-138 attenuated LPS-induced morphological changes in BV2 cells (Figure 3A). In addition, we evaluated the mRNA expression levels of iNOS, TNF-α, ARG1, and IL-10. iNOS and TNF-α are markers of M1 polarisation, while ARG1 and IL-10 are markers of M2 polarisation (Parayath et al., 2018; Zhou et al., 2021). Our results suggest that the Exos-138 group better inhibited M1 polarisation in BV2 cells compared with the Exos pre-treatment group, while contributing to the micro polarisation shift of glial cells toward the M2 phenotype (Figures 3B–E).

Furthermore, we assessed the polarization phenotype of microglia through immunofluorescence analysis. M1 microglia and M2 microglia were identified using iNOS and CD206 markers, respectively. The proportion of iNOS+/DAPI + cells was markedly elevated in the LPS group when compared to the control group. In contrast, treatment with Exos-138 significantly reduced the percentage of iNOS+/DAPI + cells in BV2 cells (Supplementary Figures S2A, B). Interestingly, we observed a significant increase in the proportion of CD206+/DAPI + cells in BV2 cells treated with Exos and Exos-138, as compared to the LPS group. Notably, the Exos-138 group exhibited a more pronounced polarization towards M2 cells, aligning with our initial expectations (Supplementary Figures S2C, D). These findings suggest that Exos-138 has the ability to modulate microglial polarization by inhibiting M1 polarization and promoting a shift towards the M2 phenotype. This indicates its potential as a therapeutic approach to mitigate neuroinflammation and promote a more favorable environment for tissue repair following SCI.

### Exos-138 inhibits LPS-induced inflammatory response in BV2 microglia

**FIGURE 4:**
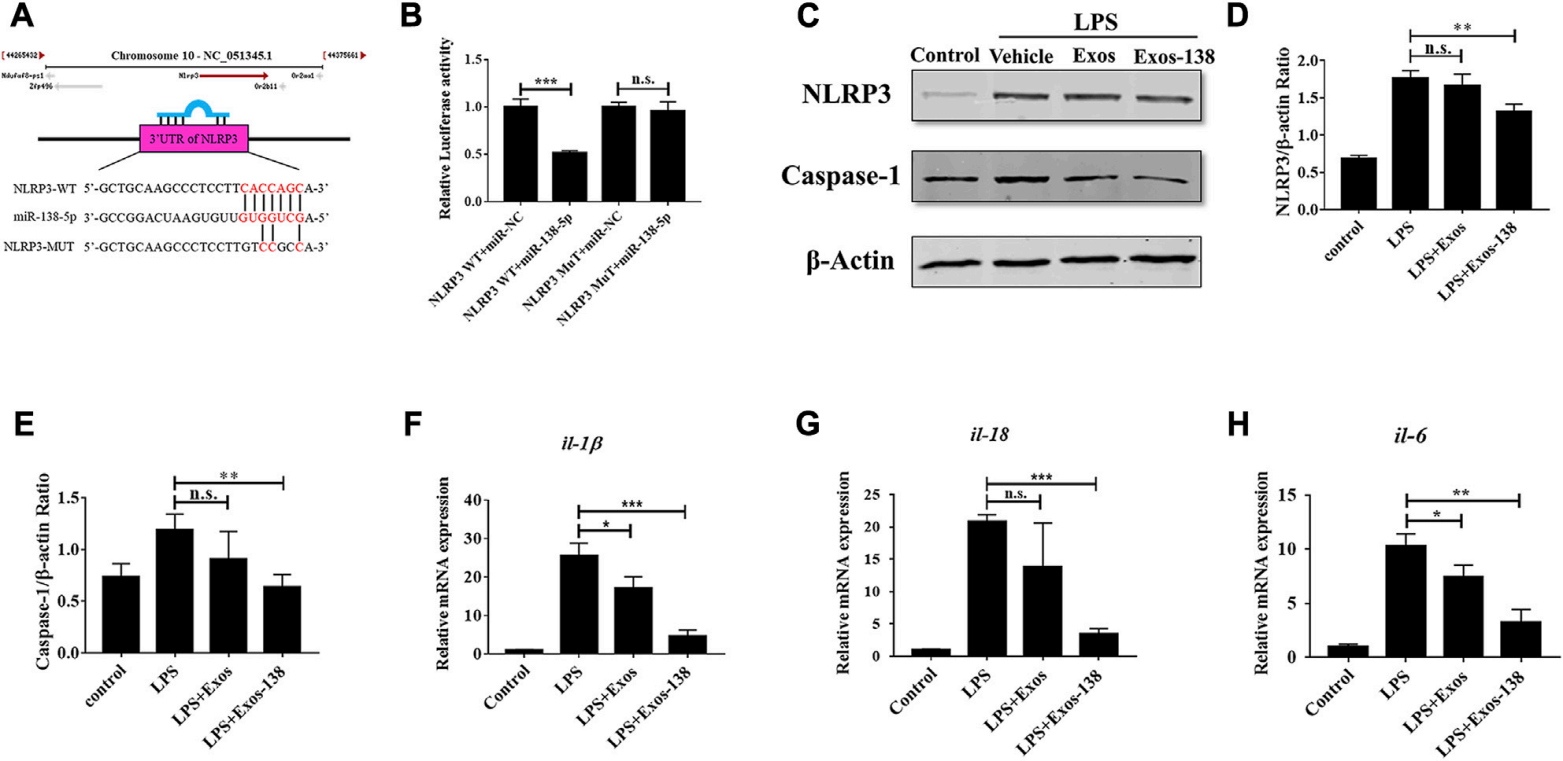
Effects of Exos-138 on LPS-induced inflammatory response in BV-2 cells. **(A)** The predicted binding sites between miR-138-5p and the 3ʹ-UTR of NLRP3. **(B)** The dual luciferase assay further verified the targeting relationship between miR-138-5p and NLRP3. **(C–E)** Representative Western blot bands and densitometric analysis of NLRP3 and Caspase-1. **(F–H)** The qRT-PCR result of *il-1β, il-18, and il-6* mRNA expression in LPS-treated BV-2 cells after pretreatment with Exos or Exos-138. The values presented are the means ± SD, *p ≤ 0.05, **p ≤ 0.01, ***p ≤ 0.001.

After SCI, neuroinflammation occurs as a necessary protective mechanism; however, excessive neuroinflammation hinders neurological regeneration (Courtine and Sofroniew, 2019). Activation of the NLRP3 inflammasome plays a key role in the poor prognosis of SCI (Mi et al., 2021). Several recent studies suggested that miR-138-5p may exert inflammatory inhibitory effects by targeting NLRP3 after CNS injury (Feng et al., 2021a; Feng et al., 2021b). In this study, we focused on the modulation of NLRP3-mediated neuroinflammation by miR-138-5p-modified engineered exosomes. To investigate the molecular mechanisms by which Exos-138 regulates microglial inflammatory responses, we further evaluated the regulatory role of Exos-138 in the NLRP3/caspase-1 signalling cascade. As shown in Figure 4A, we predicted the potential binding site of miR-138-5p in the 3ʹ-UTR of NLRP3. Consistent with previous results, the dual-luciferase reporter gene assay confirmed NLRP3 as a direct target of miR-138-5p (Figure 4B). Furthermore, we determined the protein expression level of NLRP3/caspase-1 in the LPS model of BV2 cells, and the results showed that Exos-138 significantly reduced the protein expression of NLRP3/caspase-1 compared to Exos treatment (Figures 4C–E). The qPCR experiments yielded similar results, as shown in Figures 4F–H. Compared with Exos, Exos-138 had a better anti-inflammatory effect and downregulated the mRNA expression of pro-inflammatory cytokines (IL-1β and IL-18) by regulating the NLRP3/caspase-1 signalling pathway.

In summary, our study provides valuable insights into the potential therapeutic role of Exos-138 in modulating neuroinflammation following SCI. However, further research is warranted to validate these findings *in vivo* models and clinical settings, as well as to explore additional molecular targets and mediators involved in the complex inflammatory response.

### P-Exos-138 promotes recovery of motor function after SCI in rats

**FIGURE 5:**
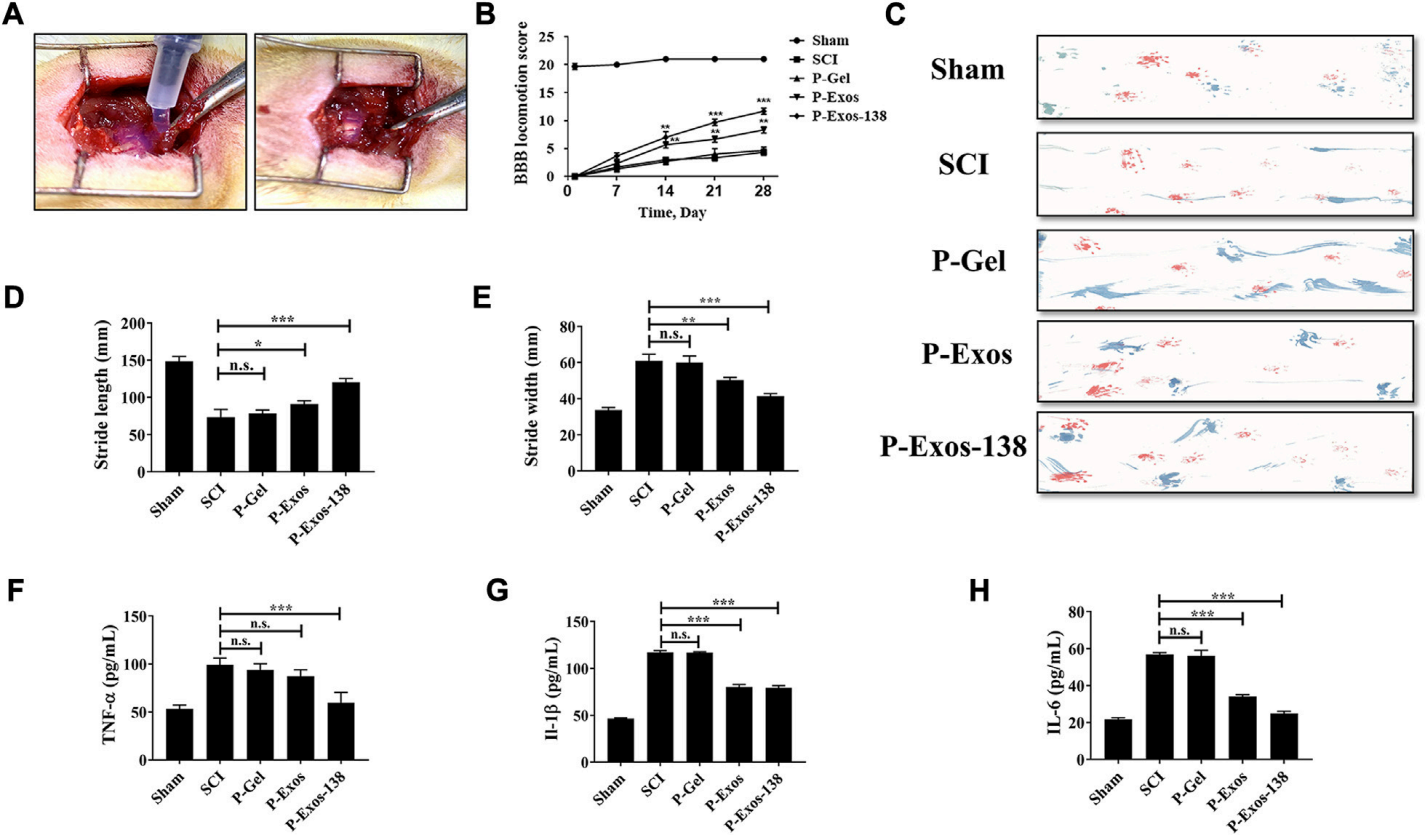
*P-Exos-138 promotes recovery of motor function after SCI i*n rats. **(A)** Schematic diagram of the novel engineered exosome delivery platform used to treat SCI rats. **(B)** BBB scores of each group at different times after spinal cord injury. **(C)** Footprint assay, representative footprints were measured on the 28th day after injury. **(D,E)** The stride length and width of different groups. **(F–H)** ELISA was used to detect the levels of pro-inflammatory mediators in the spinal cord of rats, including IL-1β, TNF-α, and IL-6. The values presented are the means ± SD, *versus* SCI group, *p ≤ 0.05, **p ≤ 0.01, ***p ≤ 0.001.

**FIGURE 6:**
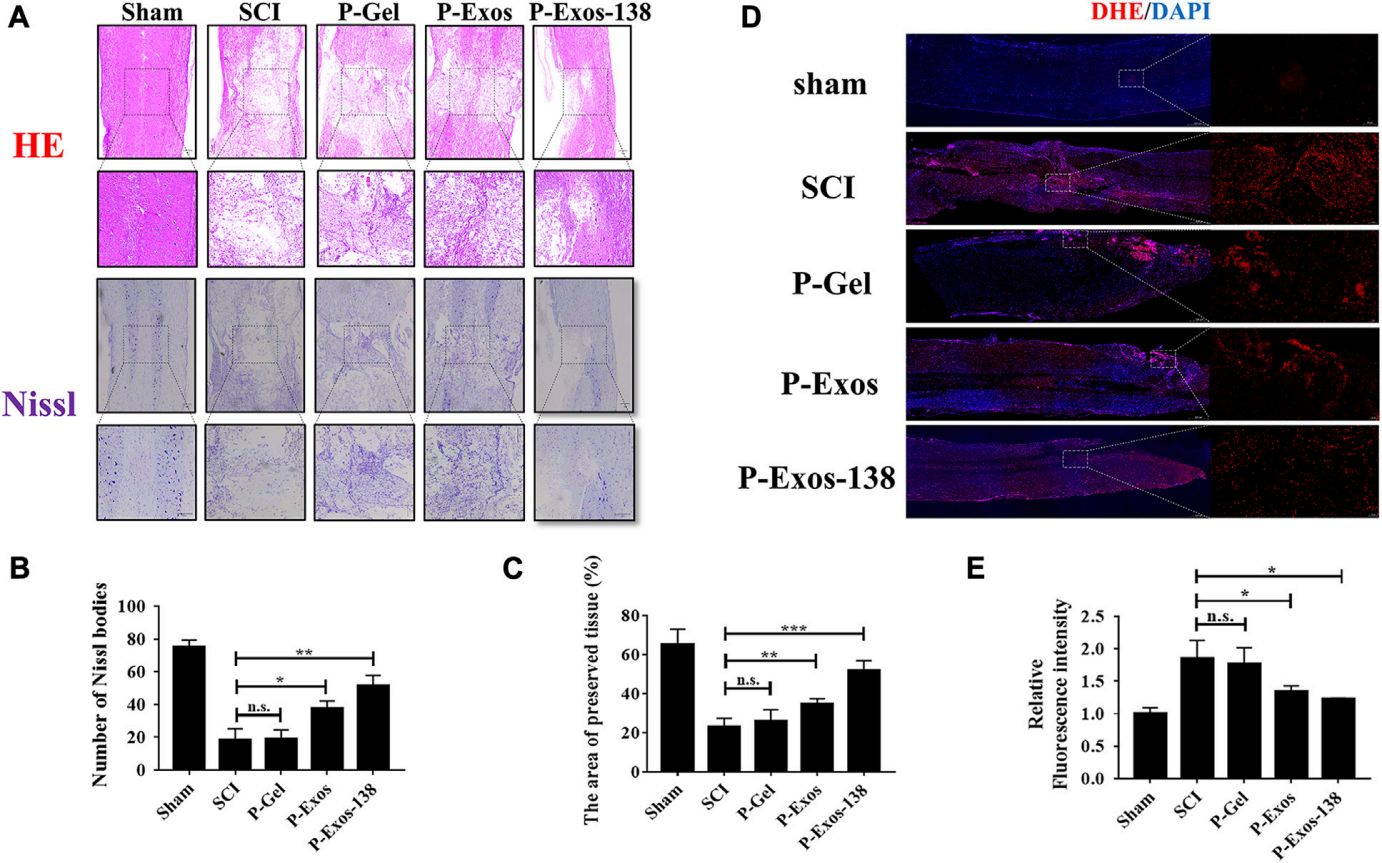
P-Exos-138 reduced histopathological injury in SCI rats. **(A)** The results of HE staining and Nissl staining of spinal cord structure of SCI rats. **(B)** Quantification analysis of the area of preserved tissues. **(C)** Quantification analysis of the number of Nissl bodies. **(D)** The Representative DHE fluorescence images of spinal cord tissue sections. **(E)** Quantification analysis of the average fluorescence intensity of DHE. The values presented are the means ± SD, *versus* SCI group, *p ≤ 0.05, **p ≤ 0.01, ***p ≤ 0.001.

In previous studies, tail vein injection was the main administration route of exosomal treatment for SCI in rats (Chen Y. et al., 2021; Jiang and Zhang, 2021). *In vivo* studies have shown that although exosomes can penetrate the blood–brain barrier, the low exosome concentration at the injury site is the main reason for their unsatisfactory therapeutic effects (Gupta et al., 2021). Recently, the application of PLGA-PEG-PLGA in CNS diseases has also been reported, including intramuscular injection of PLGA-PEG-PLGA hydrogel loaded with curcumin, which effectively prevented the development of AD in rats (Lin et al., 2022), while PLGA-PEG-PLGA hydrogel loaded with baricitinib could prevent the development of SCI by inhibiting the JAK2-STAT3 pathway in microglia at the early stage of injury, promoting recovery in rats with SCI (Zheng et al., 2021). We validated the therapeutic effects of the P-Exos-138 drug delivery system in a rat SCI model. Figure 5A shows a schematic of the use of temperature-sensitive injectable hydrogels. The BBB score was used to assess recovery after SCI in rats, and the results showed that rats in the P-Exos-138 group recovered significantly faster than those in the control group starting from 14D postoperatively (Figure 5B). Meanwhile, the rat footprint experiment reflected the improvement in locomotor ability in each group after surgery, in which the rats in the SCI and PLGA-PEG-PLGA groups showed dragging in the hind limbs, while the rats in the P-Exos and P-Exos-138 groups showed improvement in hind limb swaying (Figure 5C). In addition, the P-Exos-138 group performed better than the P-Exos group in terms of stride length and width (Figures 5D,E). Notably, we measured the levels of pro-inflammatory mediators in the spinal cord of rats 7 days after injury, and the results showed a decreasing trend of TNF-α, IL-1β, and IL-6 in the P-Exos-138 group compared to that in the SCI group (Figures 5F–H). The efficacy of P-Exos-138 was also demonstrated by the histopathological results, with less spinal cord cavity formation and an increased number of Nissl bodies in rats treated with P-Exos-138 compared to those in other treatment groups (Figures 6A–C). The above results indicate that P-Exos and P-Exos-138 could promote the recovery of motor function after SCI in rats and that P-Exos-138 had greater therapeutic effects.

While our study provides promising results regarding the therapeutic effects of P-Exos and P-Exos-138 in promoting motor function recovery and reducing inflammation in a rat SCI model, there are some limitations to consider. Future investigations should focus on exploring the underlying molecular pathways and signalling mechanisms involved in the therapeutic actions of P-Exos-138.

### P-Exos-138 regulates the microenvironment at the injury site and promotes axonal regeneration after injury

**FIGURE 7:**
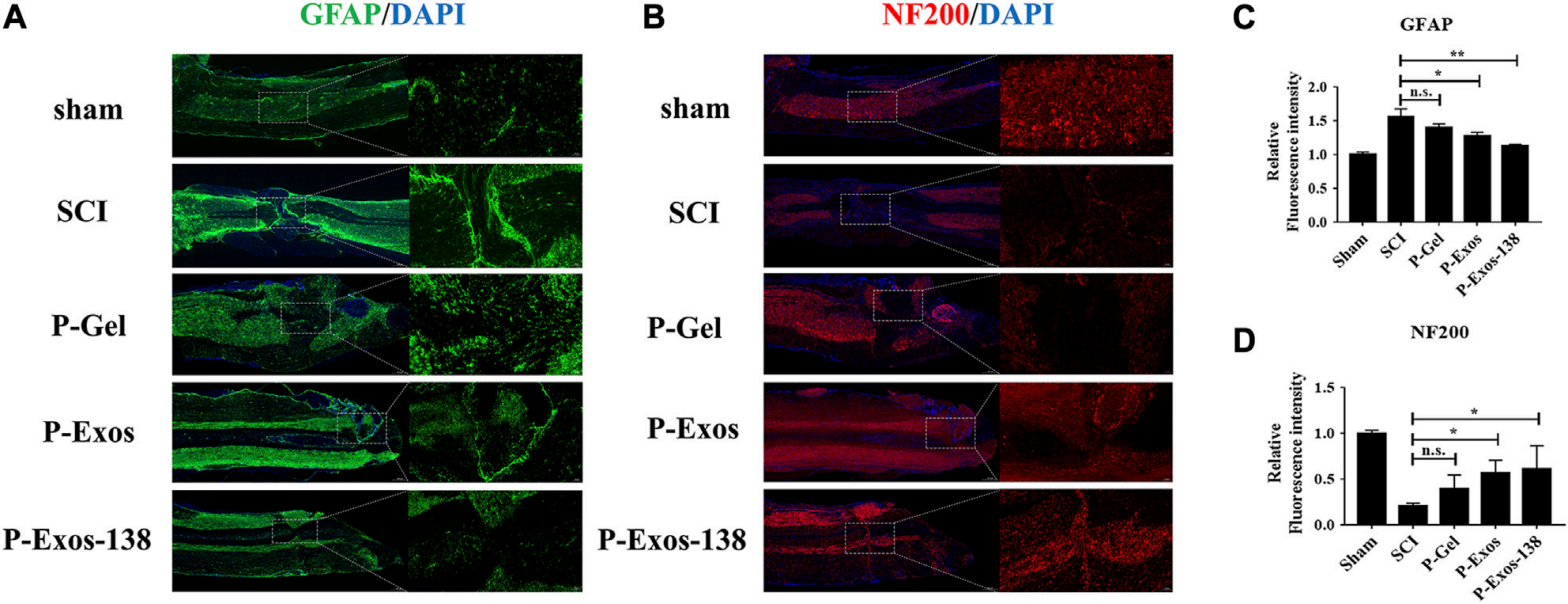
P-Exos-138 regulates the microenvironment after injury. **(A)** Representative immunostaining images of GFAP (green) in the injured site. **(B)** Representative immunostaining images of NF200 (red) in the injured areas of the spinal cord on the 28th day postinjury. **(C,D)** Quantification analysis of the average fluorescence intensity of GFAP and NF200. The values presented are the means ± SD, *versus* SCI group, *p ≤ 0.05, **p ≤ 0.01, ***p ≤ 0.001.

Oxidative stress plays an important role in the formation of the adverse microenvironment after SCI; the presence of P-Exos-138 significantly reduced ROS levels at the injury site (Figures 6D,E). Glial scarring negatively influences repair after SCI; a previous study suggested that miR-138 might inhibit astrogliosis formation by targeting vimentin (Qian et al., 2015). As shown in Figures 7A–D, we performed GFAP and NF200 immunofluorescence staining on the injury site. Our results showed that the P-Exos-138 group had a lower fluorescence intensity for GFAP-positive cells, that is, less glial scarring, distributed along the edge of the injury compared to the SCI group. In addition, NF200-labelled neurofilaments significantly increased at the injury site after P-Exos-138 treatment, indicating that P-Exos-138 contributes to axonal repair after injury.

## Conclusion

**FIGURE 8:**
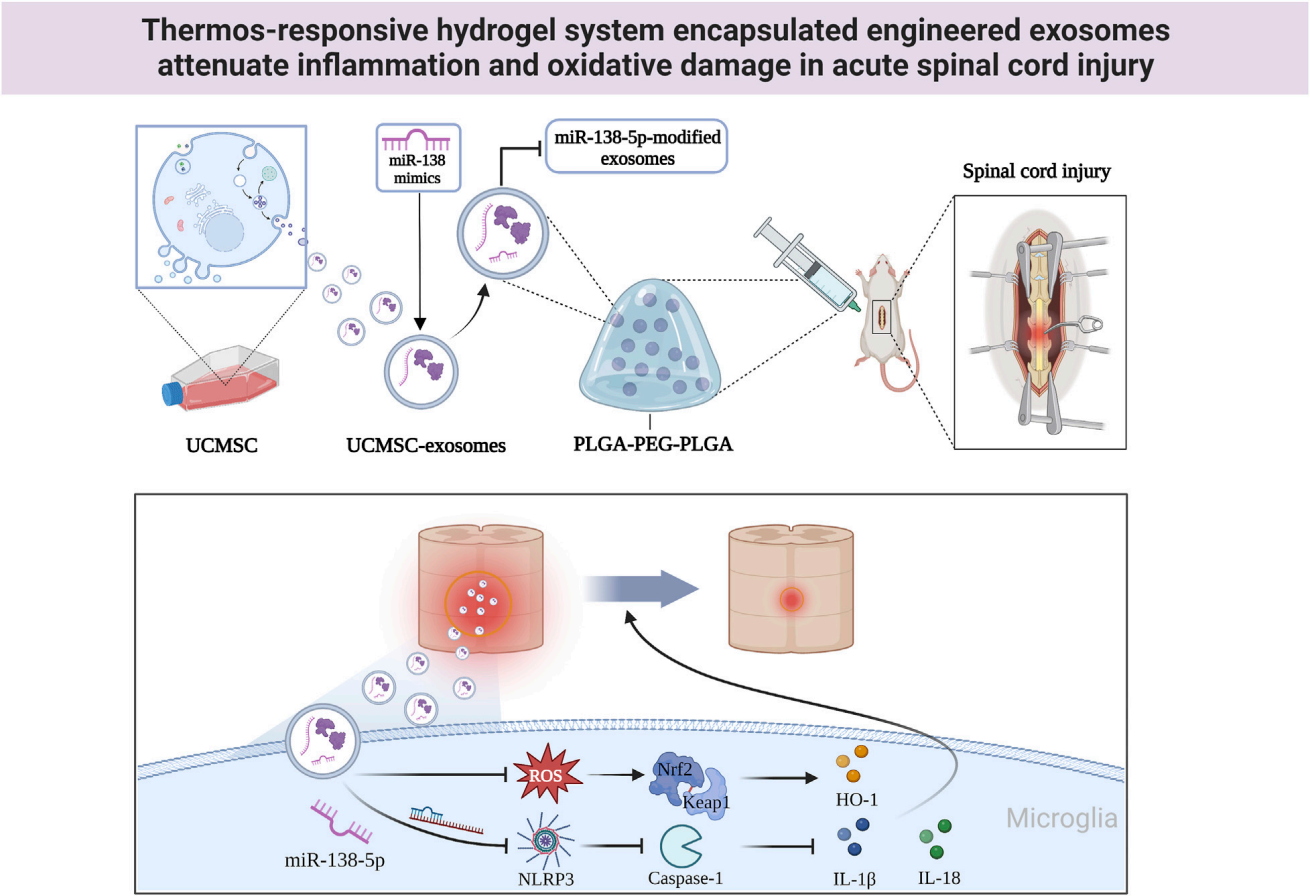
The mechanistic model of P-Exos-138 on SCI. Exos-138 treatment suppresses NLRP3/Caspase-1 inflammasome signalling pathway and oxidative damage, which protects the neurons and promotes functional recovery of SCI rats.

Overall, the novel engineered exosome delivery platform can inhibit neuroinflammation after SCI in rats by modulating the NLRP3/caspase-1 signalling cascade and Nrf2-keap1 signalling pathway to alter the activation state of microglia and reduce cellular oxidative damage. This novel exosome delivery system is a potential therapeutic strategy for the treatment of SCI (Figure 8). However, this study has several limitations. Firstly, excessive neuroinflammation is detrimental to nerve regeneration after SCI, but whether neuroinflammation is excessive remains to be explored. Secondly, the CNS is a very complex whole, and this study only focused on microglia and inflammation-related pathways, which do not provide a complete picture of the disease development process. In conclusion, our study has theoretical value, but we still need to further investigate the treatment modalities at various stages of disease development in inflammation-dependent CNS injury.

## Data Availability

The original contributions presented in the study are included in the article/Supplementary Material, further inquiries can be directed to the corresponding author.

## Supplementary material

Supplementary Figure S1 Expression value of miR-138-5p in SCI Rats. **(A)** The miR-138-5p expression in spinal cord tissue using RT-qPCR at 3 days post injure.

Supplementary Figure S2 Exos-138 inhibits LPS-induced M1 polarization of microglia. **(A-D)** Immunofluorescence staining and quantification analysis for Iba1, iNOS and CD206 in BV2 cells.
