# Supplementary figures and images for: Thermos-responsive hydrogel system encapsulated engineered exosomes attenuate inflammation and oxidative damage in acute spinal cord injury
Source: Front Bioeng Biotechnol. 2023 Aug 8;11:1216878. doi: 10.3389/fbioe.2023.1216878 (PMC10442716; doi:10.3389/fbioe.2023.1216878)

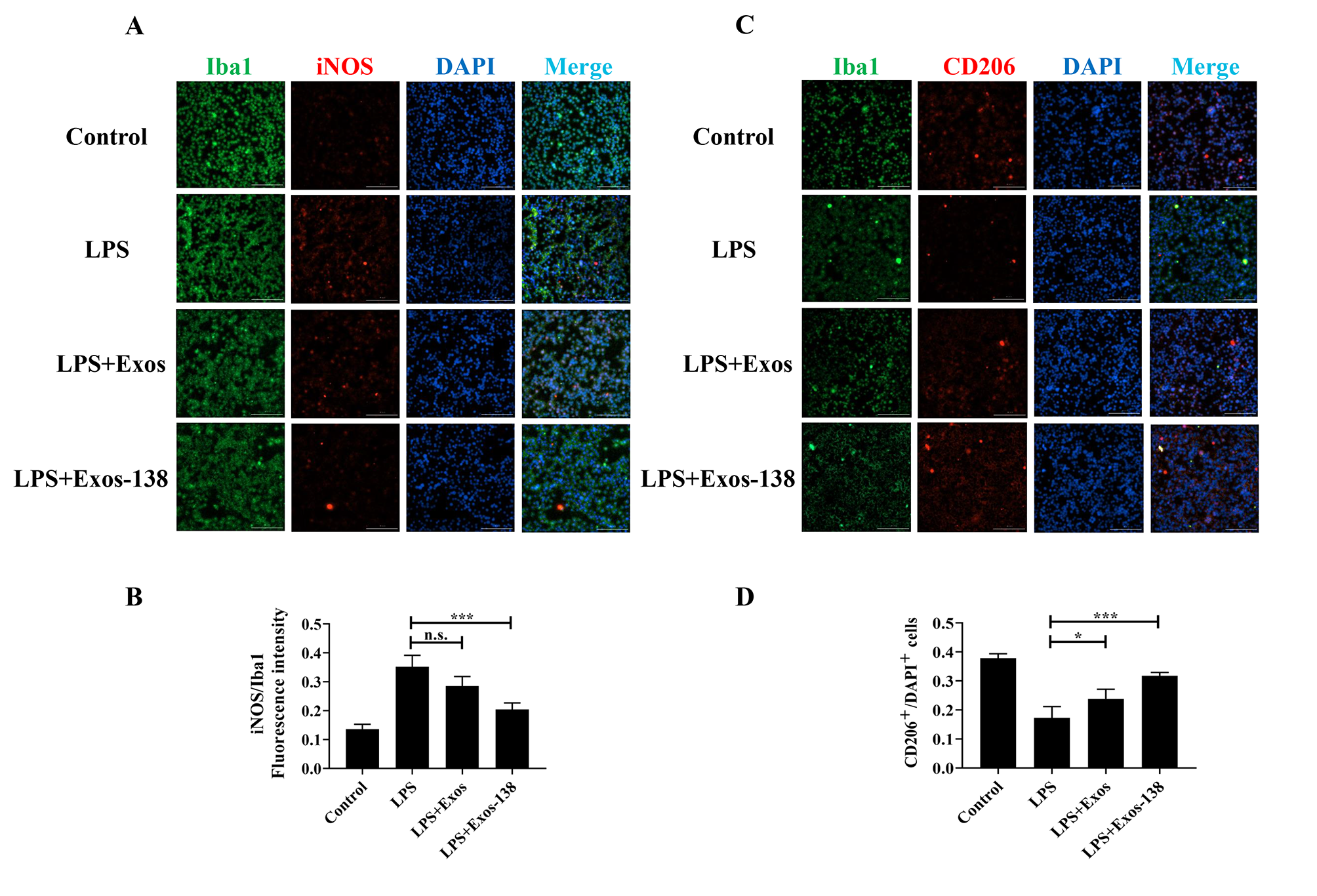

Supplement: Supplementary file 1 [file Image2.TIF]

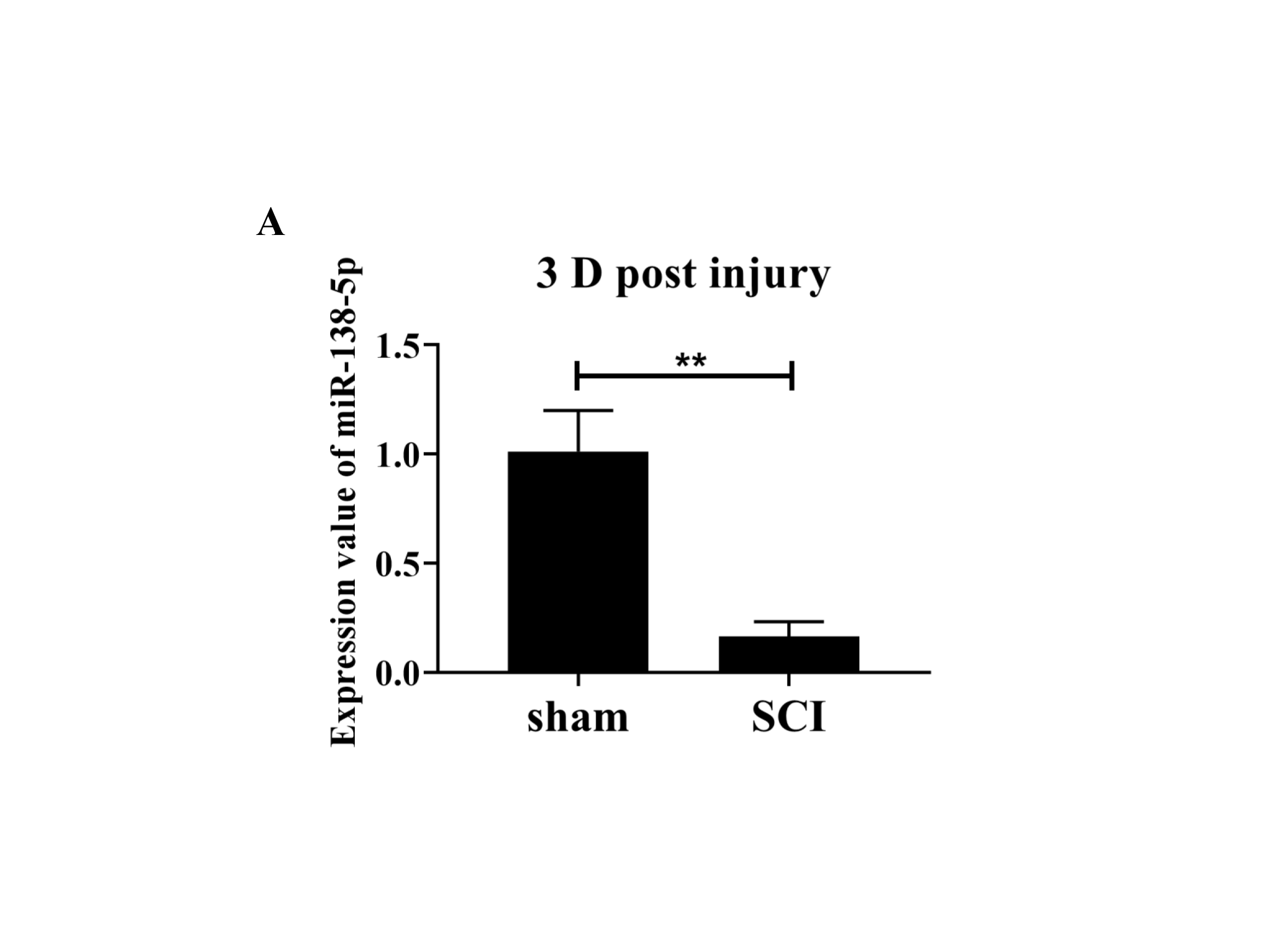

Supplement: Supplementary file 2 [file Image1.TIF]
